# Risk factors associated with postoperative delirium in elderly patients undergoing hip surgery

**DOI:** 10.3389/fpsyt.2023.1288117

**Published:** 2023-10-19

**Authors:** Xiao-Hong Liu, Qing-Fu Zhang, Ying Liu, Qing-Wang Lu, Jian-Hua Wu, Xiao-Hua Gao, Zhi-Yuan Chen

**Affiliations:** ^1^Department of Anesthesiology, the Second Affiliated Hospital of Fujian Medical University, Quanzhou, China; ^2^Department of Anesthesiology, Jinjiang Municipal Hospital (Shanghai Sixth People's Hospital Fujian Campus), Jinjiang, China; ^3^Department of Anesthesiology, Shanghai Jiaotong University Affiliated Sixth People's Hospital, Shanghai, China

**Keywords:** elderly retrospective study, hip surgery, postoperative delirium, prediction, PACU

## Abstract

**Objective:**

We retrospectively analyzed the occurrence of postoperative delirium following hip surgery and the associated risk factors. The aim was to establish a clinical foundation for preventing postoperative delirium after hip surgery.

**Methods:**

We retrospectively selected elderly patients who had hip surgery at our hospital between January 2022 and August 2022. We included patients who experienced delirium in the observation group and those who did not encounter delirium in the control group. We then proceeded to compare various indicators among these two groups of patients.

**Results:**

We analyzed a total of 97 cases of hip surgery, and among them, 32 cases experienced postoperative delirium, resulting in an incidence rate of 32.9%. Various factors were found to be linked to the development of postoperative delirium, including age, height, gender (male), preoperative erythrocyte sedimentation rate (ESR), postoperative ESR, preoperative lactate levels, pain scores on the first day after surgery, type of surgical procedure, and the occurrence of delirium in the post-anesthesia care unit (PACU delirium). Additionally, it was observed that 75% of patients who had PACU delirium also experienced postoperative delirium.

**Conclusion:**

Postoperative delirium in patients who have hip surgery had an incidence rate of 32.9%. This phenomenon is linked to various factors that pose a risk, such as the patient age, height, gender, preoperative ESR levels, postoperative ESR levels, preoperative lactate levels, pain scores on the day following surgery, and the specific surgical procedure performed. The likelihood of experiencing delirium increases by 12% for every additional 10 years in patient age. Additionally, the occurrence of delirium in the PACU is a strong indicator of the likelihood of experiencing postoperative delirium.

## Introduction

Delirium is a reversible cognitive disorder resulting from temporary damage to neurons due to an underlying systemic disturbance. This disturbance signifies an acute dysfunction in a vital organ and serves as an indicator of brain malfunction. The diagnosis of delirium is outlined in the Diagnostic and Statistical Manual of Mental Disorders, Fifth Edition (DSM-5), published by the American Psychiatric Association. This manual provides guidelines for categorizing and diagnosing mental health disorders. According to the DSM-5, delirium is characterized by rapid-onset deficits in attention, cognition, and/or consciousness that vary in severity and fluctuate over time, accompanied by a departure from usual cognitive functioning (1).

Among patients undergoing hip surgery, postoperative delirium (POD) is the most prevalent complication. Delirium also carries a substantial societal burden due to its association with heightened morbidity and mortality rates. Prolonged delirium duration further escalates the risk of postoperative mortality (2). Patients who experience POD not only encounter notably extended hospital stays but also face elevated mortality rates, reduced likelihood of returning to their pre-fracture mobility, decreased independence in daily activities, and limitations in fundamental everyday tasks for up to a year following the surgery, thus, extended period of care is necessitated (3). Hence, it is imperative to screen for risk factors associated with POD in patients undergoing hip surgery to implement measures for preventing its occurrence. Several factors that increase the likelihood of POD in elderly patients who undergo hip surgery can be categorized into two key elements: susceptibility factors and predisposing factors. Susceptibility factors mainly pertain to risk factors that are present prior to hospital admission, while predisposing factors primarily involve risk factors that arise after admission. A previous study identified advanced age, dementia, low protein levels, anemia, vision and hearing problems, low blood oxygen levels, pain, low blood pressure, and catheter usage as contributors to delirium (4). In recent times, there has been an increasing focus on POD among elderly patients, as there is a concern that POD could contribute to the development of mild cognitive impairment or even dementia (5). Older patients who are identified with mild cognitive impairment (MCI) or dementia and also experience POD have a high probability of subsequently being diagnosed with MCI or dementia (6). Patients who experience delirium after surgery exhibit a higher mortality rate, more severe health conditions, and prolonged stays within post-anesthesia care units (PACUs) and hospitals (7). POD itself is an independent risk factor for mortality during hospitalization (8). The risk factors connected to POD are diverse, and its underlying mechanisms and independent risk factors are still not fully understood. Consequently, it is imperative to investigate the factors that contribute to POD in elderly patients undergoing hip surgery, pinpointing those factors that increase the risk of POD occurrence. This will provide a theoretical foundation for preventing POD. The present study involves a retrospective analysis of medical records of patients undergoing hip surgery, aiming to outline the occurrence frequency of POD incidents and delve into the potential risk factors associated with POD in this patient cohort.

## Materials and methods

### Inclusion and exclusion criteria

Inclusion criteria were as follows: (1) patients undergoing hip surgery at our hospital; (2) patients aged >65 years, regardless of gender; (3) patients with American Society of Anesthesiologists (ASA) classification of II-III.

Exclusion criteria were as follows: (1) patients with preoperative delirium; (2) patients with preoperative cognitive deficits (illiterate <14 scores, non-illiterate <19 scores); (3) patients with a preoperative history of Alzheimer’s disease, stroke, and psychiatric disorders; (4) patients who were transferred to the intensive care unit after the surgery.

Between January 2022 and August 2022, our orthopedic division received 120 patients seeking elective hip surgery, encompassing procedures like total hip arthroplasty, femoral head replacement, and closed reduction with internal fixation for fractures. After applying specific criteria for inclusion and exclusion, a total of 97 patients met the requirements and were ultimately considered for participation in this research.

### Information collection

1. The observation group consisted of patients who experienced the onset of delirium, while the control group comprised patients who did not develop delirium.
2. Basic data was collected from every patient, encompassing details like their sex, age in years, weight in kilograms, ASA classification, presence of hypertension, diabetes mellitus, initial diagnosis before surgery, type of surgical procedure, length of surgery in minutes, duration of stay in the recovery room in minutes, occurrences of blood transfusion, utilization of self-donated blood, and outcomes of blood gas analysis.
3. We documented various factors related to the perioperative period, which included instances of delirium in the PACU, usage of the anesthetic drug remifentanil during surgery (measured in milligrams), administration of propofol in the PACU, presence of insomnia before and after surgery (on the day of surgery, the first day, and the second day after surgery), pain ratings at different time points: upon entering the operating room (T1), upon awakening after surgery (T2), on the first postoperative day (T3), and on the second postoperative day (T4). We also tracked the occurrence of adverse reactions during the perioperative period, including cases of nausea, vomiting, hypotension, hypertension, and complications related to nerve blocks (such as reactions to local anesthetic toxicity, nerve injury, local hematoma, and infection). Additionally, we monitored levels of inflammatory markers (c-reactive protein [CRP] and erythrocyte sedimentation rate [ESR]) before and after surgery, as well as blood gas parameters (pH, partial pressures of oxygen [PaO2], partial pressures of carbon dioxide [PaCO2], blood glucose, lactate, and hemoglobin) before and after surgery. The utilization of remedial analgesic drugs was also recorded in terms of the number of cases.

### Assessment criteria for delirium

The nursing delirium screening scale (Nu-DESC) was utilized to identify delirium (9). This assessment scale is straightforward, fast, and easily learnable. Delirium was determined if the overall Nu-DESC score reached 2 points or more (Table 1).

**Table 1:** Nursing delirium screening scale for the diagnosis criteria of delirium.

Nursing delirium screening scale – Symptoms	Scores
Disorientation: verbal or behavioral signs of confusion about time or place or the identity of others around them	0–2
Inappropriate behavior: the behavior of patients disproportionate to the situation and/or the status of patients, such as pulling on catheters or dressings or attempting to get out of bed when not permitted to do so and similar behavior	0–2
Illusion/hallucination: seeing or hearing non-existent things, distortion of vision	0–2
Psychomotor retardation: slow response, no or little spontaneous activity/speech. For instance, patient is unresponsive to needling and/or cannot be aroused	0–2

Delirium is diagnosed with a total score greater than or equal to 2.

### Statistical analysis

We performed statistical analysis on all the collected data using SPSS 23.0 software. Measurement data are presented as mean ± standard deviation (mean ± SD). To compare data between two groups, we employed the *t*-test, while for comparing data among three or more groups, analysis of variance (ANOVA) was used. In cases of intra-group comparisons, we utilized repeated-measures ANOVA. Counting data were analyzed using the chi-squared test (χ2) test (the corrected χ^2^ test and Fisher’s exact probability method). Statistical significance was determined at a threshold of *p* < 0.05.

## Results

**Figure 1:**
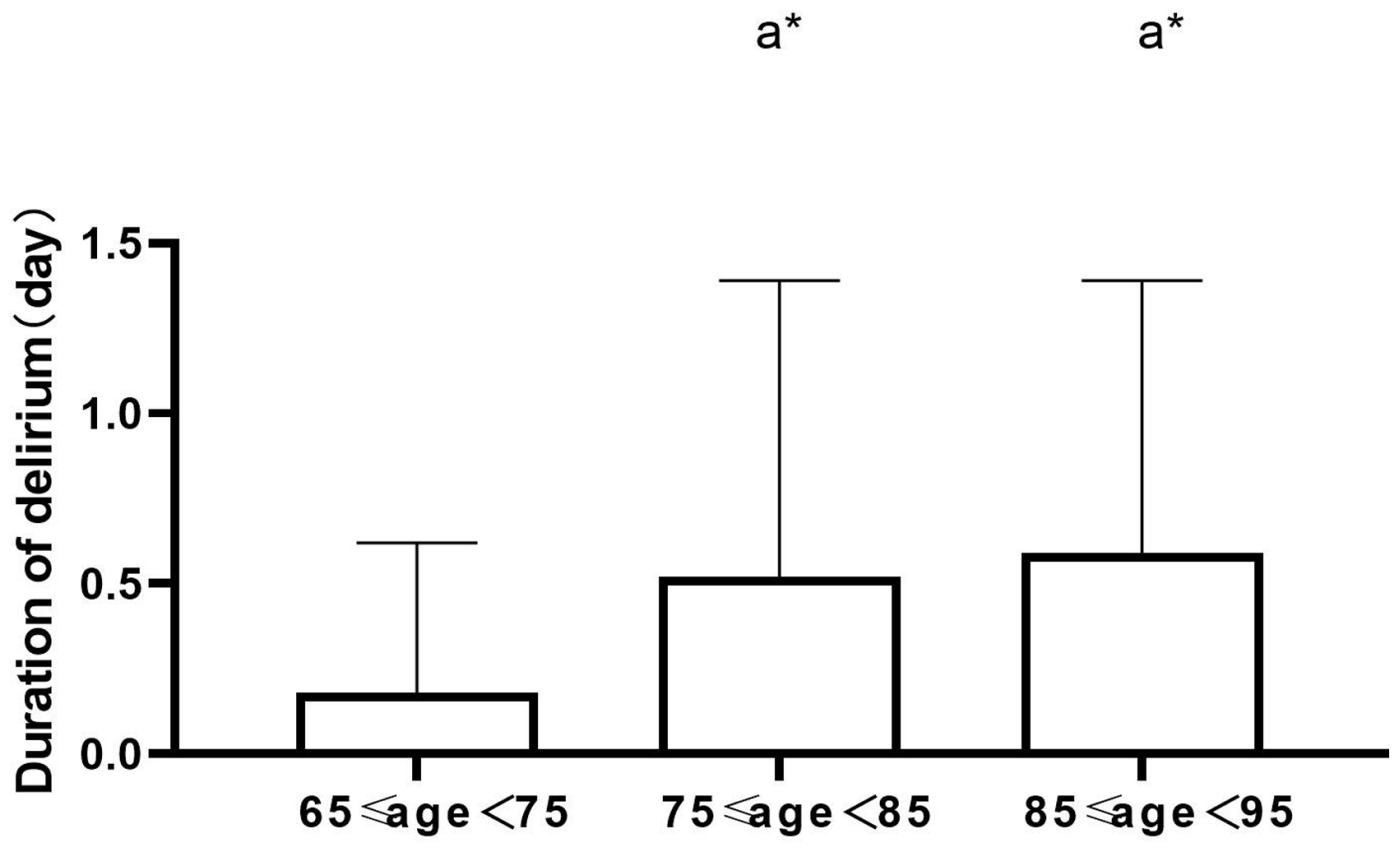
Comparisons of delirium duration by age stratification, a* *p* < 0.05.

**Figure 2:**
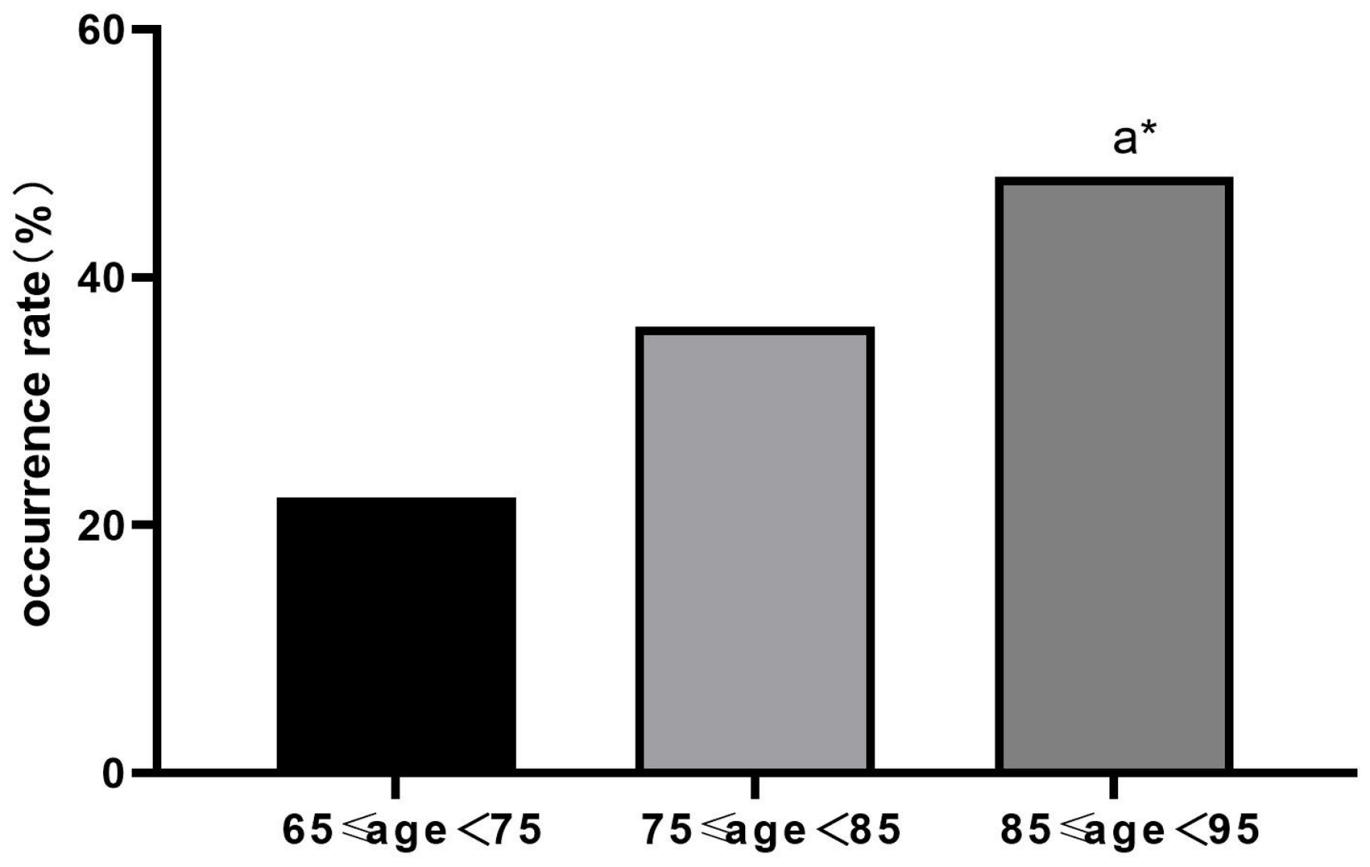
Comparisons of incidence of delirium by age stratification (%), a* *p* < 0.05.

**Figure 3:**
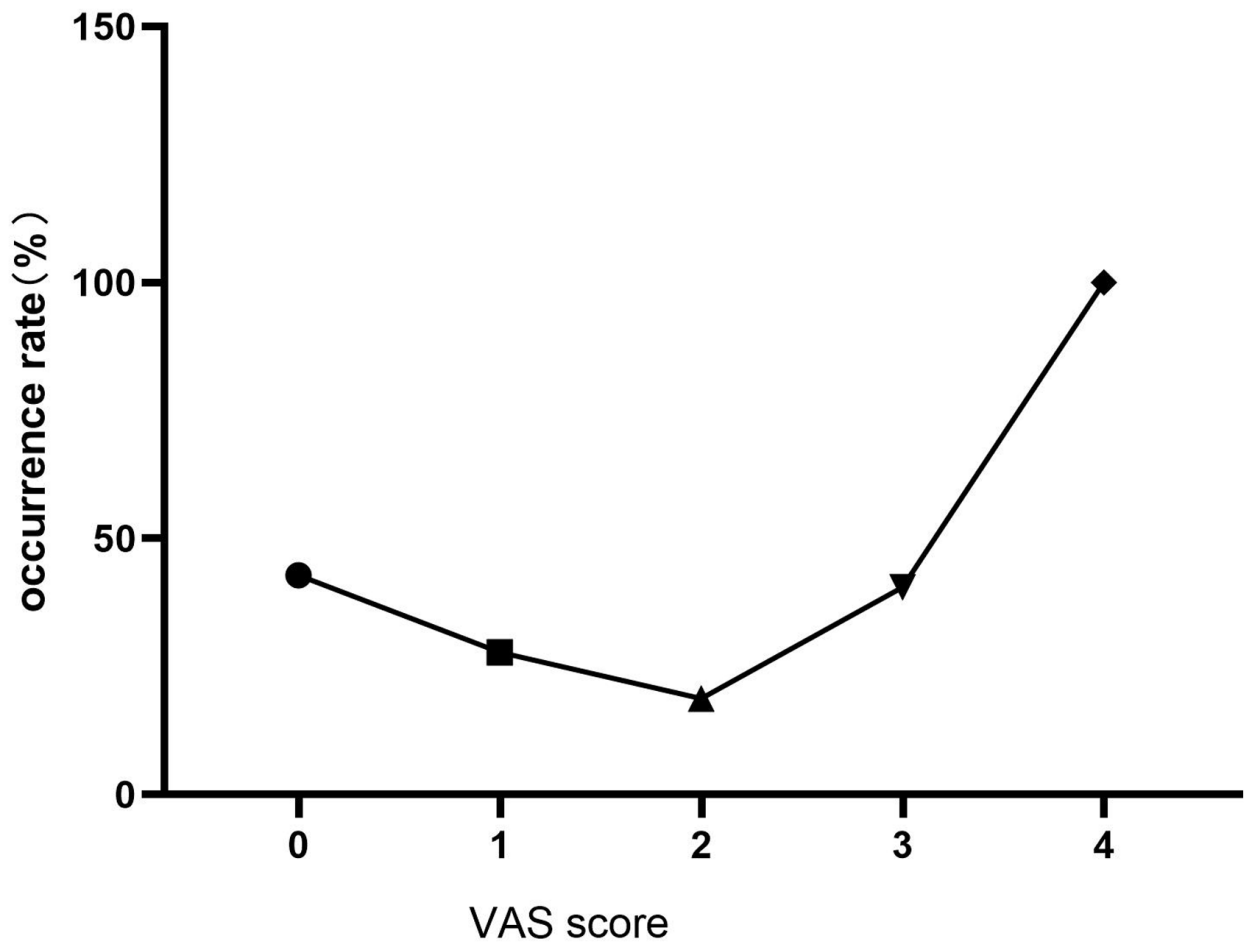
Incidence of delirium increased with elevated VAS and the difference was statistically significant (p < 0.05).

We collected and examined 43 risk factors associated with the occurrence of POD. We identified several influential risk factors for POD, including age, height, gender, preoperative and postoperative ESR levels, preoperative lactate levels, pain scores on the day following surgery, type of surgical procedure, and the presence of delirium in the PACU. These risk factors displayed significant statistical differences (*p* < 0.05). Furthermore, the frequency of delirium exhibited noteworthy variation across different surgical methods. Upon conducting pairwise comparisons, we determined that the incidence of delirium was significantly higher in the closed reduction and internal fixation group compared to the total hip arthroplasty group (*p* < 0.05). However, there was no statistically significant difference observed in other pairwise comparisons (Table 2). There was a statistically significant difference in the duration of delirium across different age subgroups (*p* < 0.05), where older age correlated with lengthier durations. Comparisons between pairs of groups revealed that the duration of POD in the 75 to 85 age subgroup was longer than in the 65 to 75 age subgroup, and the difference was statistically significant (*p* < 0.05). Similarly, the duration of POD in the 85 to 95 age subgroup was longer than in the age subgroup 65 to 75, and the difference was statistically significant (p < 0.05). However, the difference in POD duration between the 75 to 85 and 85 to 95 age subgroups was not statistically significant (*p* > 0.05). The frequency of POD increased with age and rose by 12% for every 10-year increment in age. Pairwise comparisons highlighted a significantly higher incidence of POD in the 85 to 95 age subgroup compared to the 65 to 75 age subgroup. There was no statistically significant difference for other pairwise comparisons (Tables 3, 4 and Figures 1, 2). The occurrence of POD exhibited a notable increase in correlation with higher pain scores on the initial day following the surgery (*p* < 0.05) (Table 5 and Figure 3).

**Table 2:** Relationships between related factors and POD (^−^x ± s).

Indexes		No delirium	Delirium	Statistic	*p*-value
Age		75.22 ± 8.30	79.81 ± 9.04	*t* = −0.249	0.014^*^
Height		157.37 ± 5.99	160.28 ± 7.37	*t* = −2.083	0.04^*^
Weight		53.95 ± 9.32	55.83 ± 10.80	*t* = −0.883	0.379
BMI		21.73 ± 3.16	21.63 ± 3.05	*t* = 0.157	0.875
Surgery duration		97.48 ± 37.91	99.78 ± 37.19	*t* = −0.283	0.778
Preoperative CRP		24.15 ± 24.41	28.57 ± 33.05	*t* = −0.742	0.46
Postoperative CRP		56.59 ± 40.14	72.98 ± 36.87	*t* = −1.941	0.055
Preoperative ESR		24.24 ± 19.22	34.56 ± 29.00	*t* = −2.089	0.039^*^
Postoperative ESR		37.06 ± 25.63	51.03 ± 25.04	*t* = −2.538	0.013^*^
Preoperative PH		7.44 ± 0.04	7.44 ± 0.04	*t* = −0.041	0.967
Postoperative PH		7.39 ± 0.06	7.40 ± 0.06	*t* = −0.779	0.438
Preoperative PaO2		74.20 ± 10.77	73.06 ± 11.76	*t* = 0.474	0.636
Postoperative PaO2		75.68 ± 9.63	74.50 ± 11.75	*t* = 0.526	0.6
Preoperative PaCO2		37.91 ± 4.96	37.91 ± 6.13	*t* = 0.001	0.999
Postoperative PaCO2		40.25 ± 5.33	39.31 ± 8.13	*t* = 0.678	0.5
Preoperative blood glucose		6.89 ± 1.90	6.18 ± 1.79	*t* = 1.764	0.081
Postoperative blood glucose		7.31 ± 1.88	6.98 ± 1.48	*t* = 0.866	0.389
Preoperative HGB		11.06 ± 2.14	10.18 ± 2.05	*t* = 1.928	0.057
Postoperative HGB		9.91 ± 1.88	9.15 ± 2.03	*t* = 1.823	0.071
Preoperative lactate		0.82 ± 0.48	0.63 ± 0.20	*t* = 2.200	0.03^*^
Postoperative lactate		0.95 ± 0.67	0.87 ± 0.39	*t* = 0.627	0.532
Gender	Male	12	13	*X*^2^ = 5.506	0.019^*^
	Female	53	19		
ASA	II	44	20	*X*^2^ = 0.258	0.612
	III	21	12		
Hypertension	Yes	31	14	*X*^2^ = 0.582	0.748
	No	34	18		
Diabetes mellitus	Yes	9	4	*X*^2^ = 0.033	0.855
	No	56	28		
Autologous blood	Yes	17	7	*X*^2^ = 0.211	0.646
	No	48	25		
Blood transfusion	Yes	6	5	*X*^2^ = 0.872	0.35
	No	59	27		
Catheterization	Yes	28	15	*X*^2^ = 0.125	0.723
	No	37	17		
Vasoactive drugs	Yes	15	7	*X*^2^ = 0.018	0.894
	No	50	25		
PACU analgesia	Yes	8	2	*X*^2^ = 0.322	0.57
	No	57	30		
Ward analgesia	Yes	21	7	*X*^2^ = 1.137	0.286
	No	44	25		
Remifentanil use	Yes	10	6	*X*^2^ = 0.176	0.675
	No	55	26		
Propofol use	Yes	28	11	*X*^2^ = 0.675	0.411
	No	37	21		
Preoperative insomnia	No	10	4	*X*^2^ = 0.144	0.704
	Yes	55	28		
Insomnia on the day after surgery	No	45	18	*X*^2^ = 1.587	0.208
	Yes	20	14		
Insomnia on the first day after surgery	No	59	25	*X*^2^ = 2.954	0.086
	Yes	6	7		
Insomnia on the second day after surgery	No	61	29	*X*^2^ = 0.332	0.564
	Yes	4	3		
Postoperative insomnia	No	44	17	*X*^2^ = 1.950	0.163
	Yes	21	15		
Pain scores in the PACU	< 4 points	58	30	*X*^2^ = 0.520	0.471
	≥ 4 points	7	2		
Pain scores on the first day after surgery	< 4 points	65	29	*X*^2^ = 3.549	0.034^*^
	≥ 4 points	0	3		
Preoperative diagnosis	Femoral trochanteric fractures	22	17	*X*^2^ = 3.323	0.19
	Femoral neck fractures	31	11		
	Hip arthropathy	12	4		
Surgery name	Closed reduction and internal fixation	23	18	*X*^2^ = 9.941	0.019^*^
	Total hip arthroplasty	31	9		
	Hemi-hip prosthesis replacement	11	5		
PACU delirium	Yes	5	15	*X*^2^ = 28.343	<0.001^*^
	No	66	12		

1. The difference in delirium occurrence across distinct preoperative diagnoses did not exhibit statistical significance. Pairwise comparisons demonstrated that there was no notable statistical significance between the groups.

2. The difference in delirium incidence among various surgical approaches displayed statistical significance. Pairwise comparisons indicated that the occurrence of delirium within the closed reduction and internal fixation group was considerably higher compared to the total hip arthroplasty group. However, differences in other pairwise comparisons did not demonstrate statistical significance.

3. The presence of an asterisk (*) denotes statistically significant differences.

**Table 3:** Comparisons of delirium duration by age stratification.

Indexes	Groups	POD duration	Statistic	*p*-value
Age	65 ≤ age < 75	0.18 ± 0.44	*F* = 3.850	0.025^*^
	75 ≤ age < 85	0.52 ± 0.87		
	85 ≤ age < 95	0.59 ± 0.80		

* indicates that the differences were statistically significant.

**Table 4:** Comparisons of the incidence of POD by age stratification (cases, %).

Indexes	Groups	No delirium	Delirium	Incidence of delirium	Statistic	*p*-value
Age	65 ≤ age < 75	35	10	22.22%	*X*^2^ = 5.269	0.072
	75 ≤ age < 85	16	9	36.00%		
	85 ≤ age < 95	14	13	48.15%		

There were no notable statistical difference in the occurrence of delirium among distinct age subcategories. However, when making direct comparisons, it was evident that the occurrence of delirium within the age range of 85 ≤ age < 95 was significantly greater than that within the age range of 65 ≤ age < 75. Additional direct comparisons did not reveal any statistically significant differences.

**Table 5:** Relationship between pain scores on the first day after surgery and POD (cases, %).

Indexes	Scores	No delirium	Delirium	Incidence of delirium	Statistic	*p*-value
VAS on the first day after surgery	0	4	3	42.86%	*X*^2^ = 10.513	0.033^*^
	1	13	5	27.78%		
	2	26	6	18.75%		
	3	22	15	40.54%		
	4	0	3	100.00%		

* indicates that the differences were statistically significant.

POD occurred in 75% of patients who suffered from PACU delirium while 15.4% of patients did not experience PACU delirium, and the difference was statistically significant (*p* < 0.05; Table 6).

**Table 6:** Relationship between PACU delirium and postoperative delirium (cases, %).

Indexes		Postoperative delirium		Incidence of delirium	Statistic	*p*-value
		No	Yes			
PACU delirium	No	66	12	15.4%	*X*^2^ = 28.343	<0.001^*^
	Yes	5	15	75.0%		

* indicates that the differences were statistically significant.

## Discussion

### Incidence of POD in elderly patients with hip fractures

According to reports, POD occurs in 4 to 53% of patients who undergo hip surgery (10). In our study, we examined a group of 97 patients who underwent hip surgery, and out of these, 32 patients (32.9%) experienced POD, a rate that aligns with previously documented occurrences. Among elderly patients, POD stands out as a common complication after hip surgery, impacting patient prognosis and cognitive function during the recovery period. It is important for medical professionals to implement preventive strategies and early identification, and intervention measures to support effective postoperative rehabilitation following hip surgery.

### Risk factors for POD in elderly patients with hip fractures

A previous study demonstrated that preoperative cognitive decline, reduced levels of intelligence, and the presence of dementia were risk factors for POD (11). In light of this, we excluded patients who exhibited cognitive impairment during the preoperative assessment, determined by their scores on the Mini-Mental State Examination (illiterate <14 scores and non-literate <19 scores). Additionally, older patients with impaired vision and hearing, as confirmed in previous research, were purposely left out of our study due to their heightened susceptibility to POD (12).

When examining the potential risk factors for delirium, our analysis revealed that age, height, being male, preoperative ESR, postoperative ESR, preoperative lactate levels, pain scores during the initial day following surgery, the type of surgical procedure, and instances of delirium in the PACU were all associated with an increased risk of developing POD. These findings are consistent with prior studies. The elderly population often contends with multiple underlying health conditions, and these preoperative comorbidities, such as hypertension and diabetes mellitus, were highlighted as contributors to the risk of POD (13). Nonetheless, our investigation did not detect any statistically significant differences in the prevalence of hypertension (36.1% vs. 34.6%) or diabetes mellitus (30.8% vs. 33.3%) between patients who developed POD and those who did not. As a result, we are unable to firmly establish a connection between diabetes mellitus, hypertension, and the onset of POD.

An earlier study had indicated a potential link between delirium and the inflammatory marker CRP (14). Nevertheless, our own study did not find any associations between preoperative or postoperative CRP levels and the occurrence of delirium. Similarly, there were no statistically significant differences noted in variables such as the duration of surgery, the need for blood transfusions, the utilization of autologous blood, the use of catheterization, or the preoperative diagnosis between the two groups under investigation.

Elderly age constitutes an autonomous risk element for POD (14). In our study, we conducted stratified analysis based on age. Based on the results of the analysis, the incidence rate of delirium was 22.22% in the 65 to 75 group age subgroup, 36% in the 75 to 85 age subgroup, and 48.15% in the 85 to 95 age subgroup. This revealed a substantial increase in the incidence and duration of delirium with advancing age. Additionally, the likelihood of delirium increased by 12% for each 10-year increase in age, in line with earlier research (15).

Heightened postoperative pain scores represent a risk factor to the occurrence of POD (16), thus ensuring effective pain management is a critical strategy for preventing POD (17). In our study, we conducted a stratified analysis of pain scores from the initial day post-surgery and found a direct correlation between increased pain scores on that day and an elevated occurrence of delirium. We investigated the connection between PACU delirium and POD. The findings indicated that 75% of patients who encountered PACU delirium also went on to develop POD, while 15.4% of patients who did not experience PACU delirium still developed POD. These findings underscore the significance of PACU delirium as a strong indicator for the onset of POD. This outcome aligns with the observations made in the study conducted by Gutiérrez et al. (18).

## Conclusion

In brief, the occurrence of POD was observed in 32.9% of elderly patients who underwent hip surgery. Factors contributing to the development of POD encompassed age, height, gender, preoperative ESR, postoperative ESR, preoperative lactate, pain scores on the day following surgery, and the specific surgical procedure undertaken. Notably, the occurrence of delirium in the PACU strongly indicated a likelihood of POD.

However, there are notable limitations and deficiencies in our study. Firstly, the participants were exclusively drawn from a single medical center, introducing the possibility of selection bias. Secondly, the number of cases examined was inadequate, with a relatively small subset of patients experiencing POD. Thirdly, the scope of collected data was limited. Specifically, we only collected information regarding patients with hypertension and diabetes mellitus, omitting details about disease management. These limitations underscore the need for further investigations aimed at analyzing the causes and risk factors contributing to the onset of POD in patients undergoing hip surgery.

## Data availability statement

The original contributions presented in the study are included in the article/supplementary material, further inquiries can be directed to the corresponding authors.

## Ethics statement

The study was conducted in accordance with the Declaration of Helsinki. The study was approved by the Ethics Committee of the Second Affiliated Hospital of Fujian Medical University. Written informed consent was obtained from all participants.

## Author contributions

X-HL: Data curation, Formal analysis, Investigation, Visualization, Writing – original draft, Writing – review & editing. Q-FZ: Conceptualization, Formal analysis, Resources, Software, Visualization, Writing – review & editing. YL: Data curation, Formal analysis, Project administration, Software, Writing – review & editing. Q-WL: Investigation, Resources, Supervision, Writing – review & editing. J-HW: Conceptualization, Data curation, Investigation, Validation, Visualization, Writing – review & editing. X-HG: Conceptualization, Data curation, Methodology, Resources, Writing – original draft, Writing – review & editing. Z-YC: Formal analysis, Funding acquisition, Resources, Software, Supervision, Writing – original draft, Writing – review & editing.
